# A High Power-Density, Mediator-Free, Microfluidic Biophotovoltaic Device for Cyanobacterial Cells

**DOI:** 10.1002/aenm.201401299

**Published:** 2014-09-16

**Authors:** Paolo Bombelli, Thomas Müller, Therese W Herling, Christopher J Howe, Tuomas P J Knowles

**Affiliations:** Department of Biochemistry, University of Cambridge, Tennis Court RoadCambridge, CB2 1QW, UK; Department of Chemistry, University of Cambridge, Lensfield RoadCambridge, CB2 1EW, UK

**Keywords:** biophotovoltaic devices, microfluidics, bioenergy, cyanobacteria

## Abstract

Biophotovoltaics has emerged as a promising technology for generating renewable energy because it relies on living organisms as inexpensive, self-repairing, and readily available catalysts to produce electricity from an abundant resource: sunlight. The efficiency of biophotovoltaic cells, however, has remained significantly lower than that achievable through synthetic materials. Here, a platform is devised to harness the large power densities afforded by miniaturized geometries. To this effect, a soft-lithography approach is developed for the fabrication of microfluidic biophotovoltaic devices that do not require membranes or mediators. *Synechocystis sp.* PCC 6803 cells are injected and allowed to settle on the anode, permitting the physical proximity between cells and electrode required for mediator-free operation. Power densities of above 100 mW m^-2^ are demonstrated for a chlorophyll concentration of 100 μM under white light, which is a high value for biophotovoltaic devices without extrinsic supply of additional energy.

## 1. Introduction

Fuelling the ever-growing need for energy^1^ by fossil combustibles is expected to have dramatic, global consequences on climate and ecosystems. These environmental effects, in combination with the depletion of fossil fuel reserves, have led to a pressing need for developing technologies for harnessing renewable energy.^2^,^3^ In this scenario, bio-electrochemical systems, such as microbial fuel cells^4^–^7^ (MFCs) and biological photovoltaic cells^8^–^12^ (BPVs), may help to alleviate the present concerns by utilizing living organisms as inexpensive, readily available catalysts to generate electricity. A particularly advantageous feature of BPVs is that they consist of living photosynthetic material that allows for continuous repair of photodamage to key proteins.

Whereas MFCs use heterotrophic bacteria to convert the chemical energy stored in organic matter, BPVs use photosynthetic organisms capable of harnessing solar energy. In MFCs operating with *Geobacter sulfurreducens*, the oxidation of acetate can proceed with a Coulombic efficiency of nearly 100%.^13^ Nevertheless, the availability of acetate and other organic substrates is not endless, which imposes a limiting factor to this approach. By contrast, in BPV-type systems, the conversion efficiencies of light into charges remain low (≈0.1%),^14^ but the primary fuel (i.e., solar light) is virtually unlimited. Consequently, a significant research effort is required towards understanding which processes limit the performance of biophotovoltaic cells, both in terms of biophysics and engineering.

In this context, miniaturization of BPVs provides highly attractive possibilities for high-throughput studies of small cell cultures, down to individual cells, in order to learn about differences in genetically identical organisms as well as to direct the evolution of efficient cell lines in bulk^15^–^17^ and in microfluidics.^18^ Furthermore, the distances that the charge carriers have to migrate within the devices can be shortened dramatically, reducing resistive losses in the electrolyte.^4^ The readily achievable conditions for laminar flow and sessile state of the anodophilic photosynthetic cells also permit operation without the use of a proton-exchange membrane.^19^–^22^

To date, efforts have focussed on miniaturized microbial fuel cells.^7^,^22^–^30^ In order to exploit the high power densities available through the decrease of the length scales of the charge transport and the decrease of the electrolyte volume, we have developed a simple fabrication method for microfluidic biophotovoltaic (μBPV) devices^23^ that do not require an electron mediator or a proton-exchange membrane. Besides increasing efficiency and simplicity of the device, relinquishing mediator and membrane also reduces the cost of potential large-scale applications.^14^,^31^–^33^

**Figure 1:**
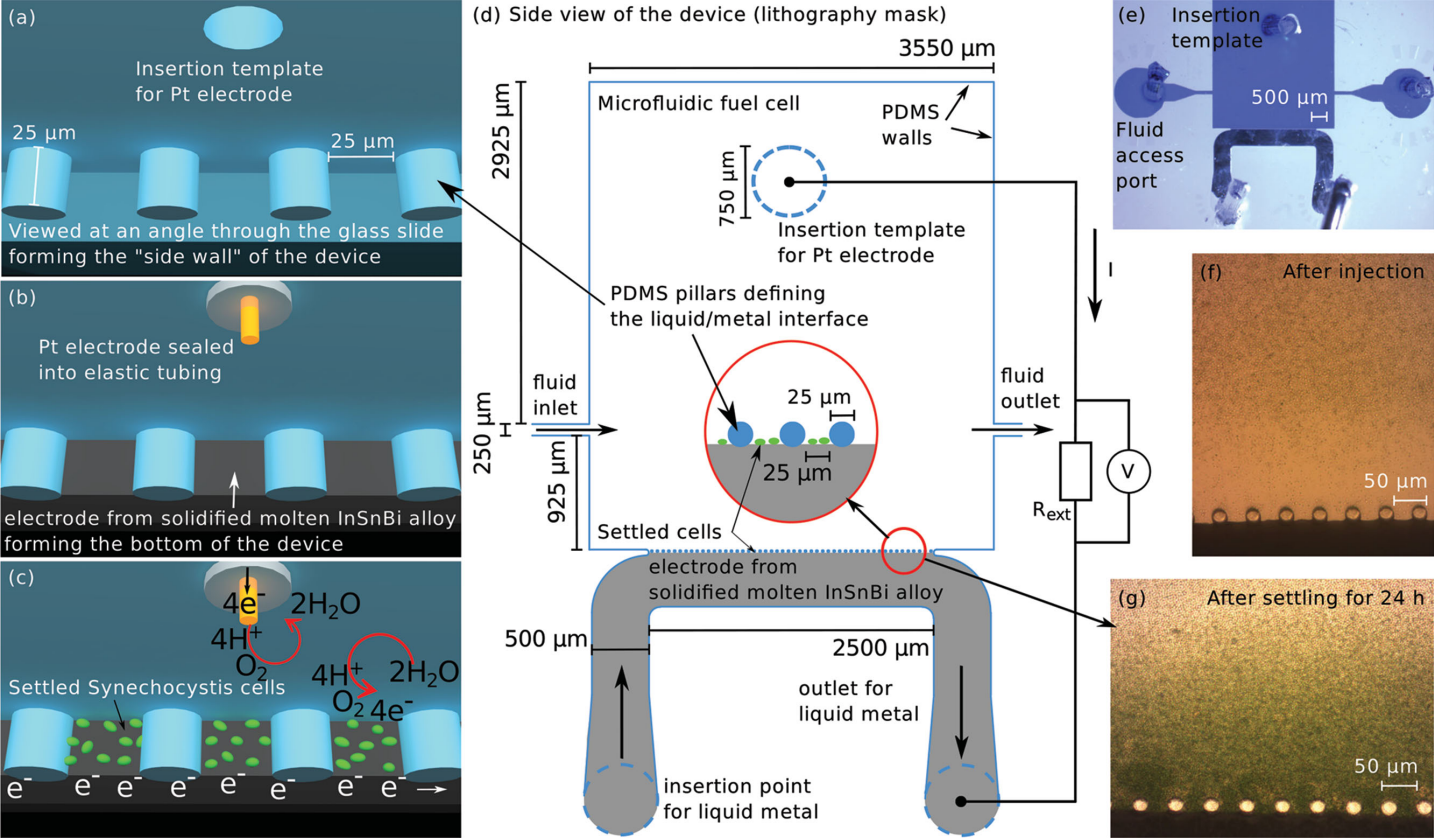
a) Schematic of the device before insertion of the electrodes, seen at an angle through the glass slide. The lithographically defined PDMS pillars retain molten metal due to its surface tension, and the hole provides an opening for insertion of the Pt electrode. b) Model of the full device including platinum cathode and InBiSn anode. c) Schematic representation of the microfluidic biophotovoltaic device in action. *Synechocystis* cells settled by gravity on the InBiSn electrode deliver electrons to the latter by oxidizing water. On the platinum cathode oxygen and hydrogen ions are supplied with electrons and combine to water, which closes the circuit. d) Top view of the device design. e) True-color image of a device filled with a solution containing Coomassie blue to visualize the 25 μm high channels. f) True-color image of a device immediately after injection of Synechocystis cells at a chlorophyll concentration of around 100 μM. g) True-color image of a device filled with *Synechocystis* cells that were allowed to settle on the anode during 24 h.

We use soft lithography^34^ to form microscopic channels which we equip using microsolidics^35^ with a self-aligned electrode from a low-melting point alloy^36^–^38^ (InSnBi) and a platinum electrode sealed inside microfluidic tubing. A scheme of such a device is shown in **Figure** **1**a–c, and the specific design including the external measurement circuit is presented in Figure 1d. True-color microscopy photographs of a device filled with Coomassie blue, with freshly injected *Synechocystis* cell, as well as with cells that have settled on the anode during 24 h are shown in Figure 1e–g, respectively. The possibility of omitting the mediator arises from the physical proximity of the settled cells and the anode which forms the bottom of the device, as well as the choice of electrode materials. The latter ensures that H^+^ is preferably reduced at the cathode since platinum catalyses this reaction.

The inherently small size (below 400 nL) of our microfluidic approach permits studies of minute amounts of biological material. Moreover, our μBPV works without any additional energy supply, such as inert gas purging to keep the anodic chamber anoxic and/or oxygen gas purging in the cathodic chamber to facilitate the reformation of water,^8^,^39^,^40^ or a bias potential applied to polarize the electrodes and improve the electron flux between anode and cathode.^33^

The use of soft lithography allows for fast in-house prototyping and for the utilization of the range of techniques developed for integrated circuits. Despite the small volumes contained in microfluidic devices, such approaches can be scaled up by parallelization,^30^,^41^ and the surface-to-volume ratio can be designed to outperform macroscopic approaches significantly.^29^

## 2. Results

The microfluidic BPV device described here operates as a microbial fuel cell with submicroliter volume, generating electrical power by harnessing the photosynthetic and metabolic activity of biological material. Its anodic half-cell consists of sessile *Synechocystis* cells, performing water photolysis (2H_2_O → 4H^+^ + 4e^−^ + O_2_) and subsequent “dark” metabolism, as well as an anode made from an InSnBi alloy and a light source.

### 2. 1. Current and Power Analyses

A μBPV was loaded with wild type *Synechocystis sp.* PCC 6803 cells (subsequently referred to as *Synechocystis*) suspended in BG11 medium supplemented with NaCl at a final chlorophyll concentration of 100 nmol Chl mL^−1^. The exo-electrogenic activity of three biological replicates of sessile cells was characterized under controlled temperature conditions sequentially in the same device.

**Figure 2:**
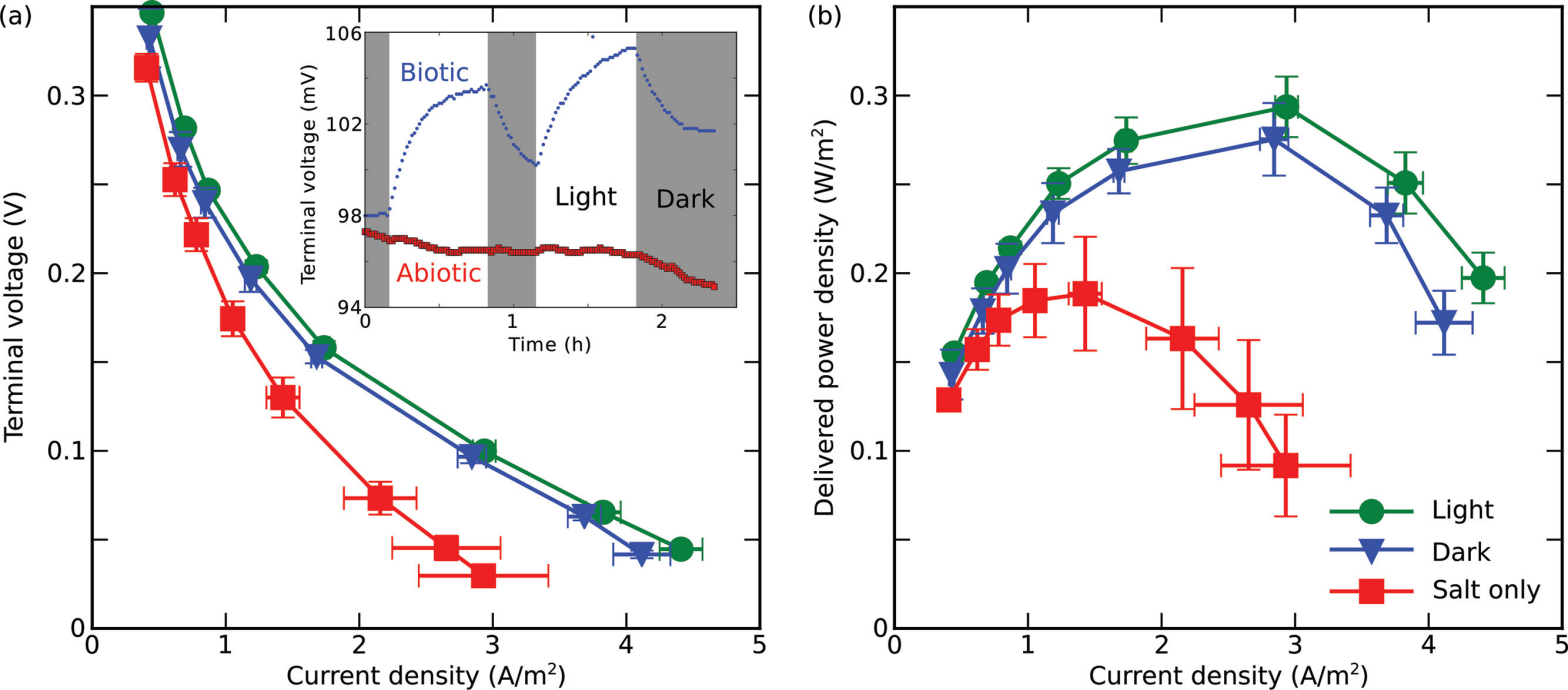
a) Comparison of the voltage output from the same microfluidic device loaded with salt medium only (BG11) or Syenechocystis cells in medium in the dark and with light. The *x*-axis has been converted to a current density through division of the measured current by the surface of the InSnBi anode, and the error bars show the standard deviations for three consecutive, independent repeats on the same device. Inset: Response of a biophotovoltaic device as well as of an abiotic control under sequential illumination. b) Power density generated by the microfluidic devices filled with salt or cells in dark/illuminated environment.

The μBPV was rested for 24 h, permitting the formation of cellular films on the anodic surface and stabilizing the open circuit potential. Polarization and power curves were then recorded by connecting different resistance loads to the external circuit in the dark or under illumination with a white light-emitting diode (LED) (see Experimental Section) and are shown in **Figure** **2**.

In the dark, significant power output was observed relative to the control sample containing no cells. This observation is consistent with the breakdown of stored carbon intermediates accumulated during the light period.^11^ The peak power output of 275 ± 20 mW m^−2^ was established at a current density of 2840 ± 110 mA m^−2^. Under illumination the microfluidic BPV loaded with *Synechocystis* showed an increase in both current and power output. The peak power density was *P*/*A* = 294 ± 17 mW m^−2^ established at a current of 2940 ± 85 mA m^−2^. Crucially, both the dark and the light electrical outputs were significantly higher than the abiotic peak power output in this device of 189 ± 32 mW m^−2^ established at a current of 1430 ± 120 mA m^−2^, demonstrating that the power output from our devices originates from the biological activity of the cyanobacteria.

From the linear slope at the high current side of the polarization curve as well as the from the external resistance for which maximal power transfer occurs we can estimate the internal resistance of the device to be around 2.2 MΩ for the biotically loaded device and 1.4 MΩ for the abiotic control (for further details see Supporting Information).

The electrical output recorded from the abiotic control - possibly due to medium salinity^5^,^42^ and anodic oxidation - is taken into account when the power densities of biotic experiments are quoted. Specifically, subtracting the abiotic background yields a biotic output power density of 105 mW m^−2^. This number is halved when comparing to the full cross-sectional area of the device (including the inaccessible parts of the anode), and the power available per footprint area is ca. 50 μW m^−2^.

### 2. 2. Light Response

To demonstrate the photoactivity of the *Synechocystis* cells, the variation of the anode-cathode voltage as a response to repeated light stimulation was recorded over time (see inset of Figure 2a). The external resistor was fixed at 100 MΩ, and the voltage was sampled once per minute. Illumination by white LED light at 200 μmol m^−2^ s^−1^ resulted in a reproducible voltage increase at a rate of 21.7 ± 4.7 mV h^−1^ with Δ*V*_light-dark_ = 5.2 ± 0.6 mV. The time until the electrical outputs were stabilized was around 1 h. We find that the baseline voltage levels change after illumination, most certainly due to a buildup and breakdown of intracellular metabolites.

From the measured spectrum of the light source (see Supporting Information) we can determine the average wave number which corresponds to a wavelength of 570 nm. Thus the photon flux can be converted to an incident light intensity of 42 W m^−2^. Using these values we can extract a rough estimate for the efficiency of our BPV (energy output versus energy input) of around 0.25%, which compares favorably to previously achieved values.^14^,^23^,^43^ Note that light scattering on the glass surface and losses from the non-perpendicular illumination angle would increase this number and hence it can be understood as a lower bound.

With such an illumination cycle, the light-driven electrical response of a device can be directly compared to dark conditions, proving the functionality of our μBPV. In addition, the abiotic control shows no variations in anode-cathode potential under similar illumination.

The difference between the power outputs under dark and illuminated conditions is consistent with previous studies of *Synechocystis sp.* PCC 6803.^14^ Nevertheless, a direct comparison of the power output reported by McCormick et al. of around 0.12 mW m^−2^ with the peak value in excess of 100 mW m^−2^ demonstrated here emphasizes the great potential of microfluidic approaches compared to macroscopic devices.

### 2. 3. Variability of the Abiotic Characterization

In order to characterize the variability of the electrical behavior of our μBPV, two further, lithographically identical devices were studied with abiotic loading (i.e., without photosynthetic cells). These devices were injected with BG11 media (with 0.25 M NaCl), and the current and power outputs were characterized under controlled temperature conditions.

**Figure 3:**
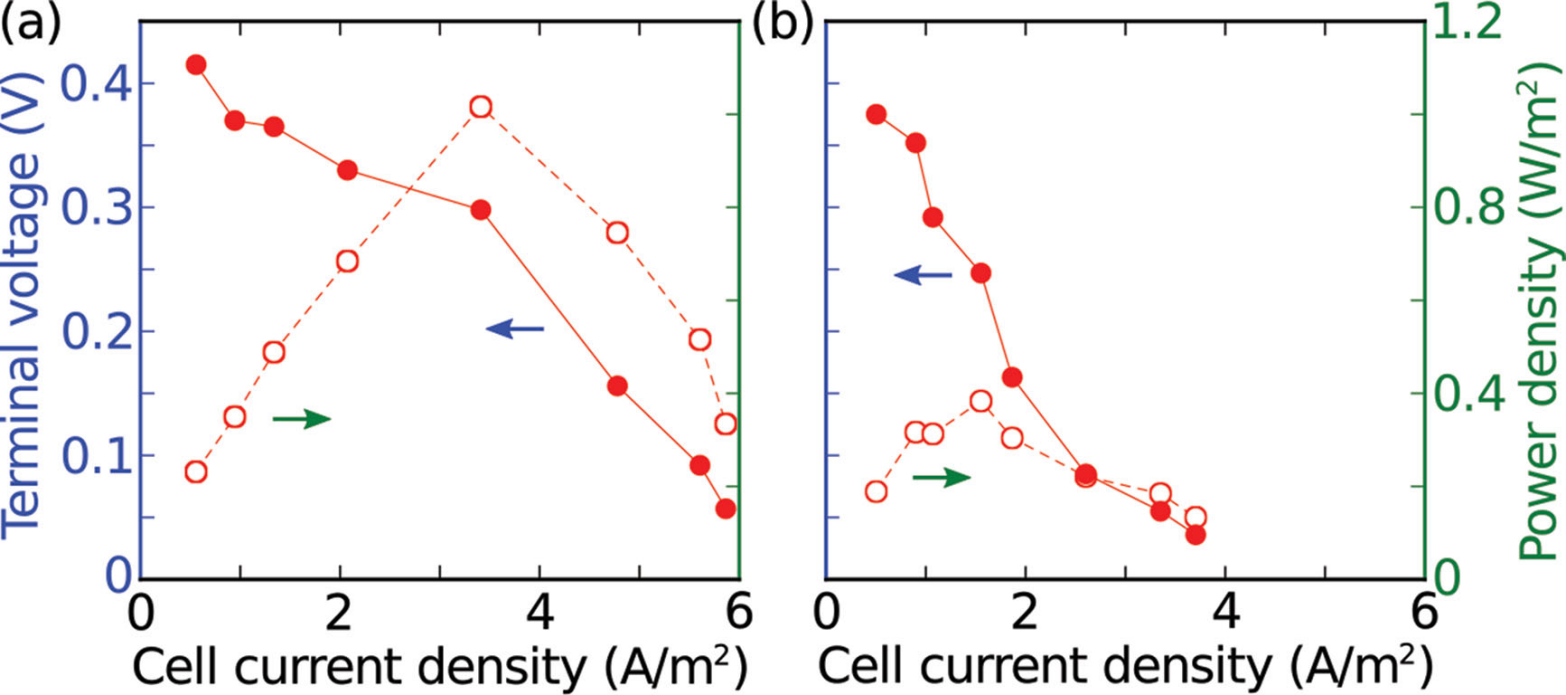
a,b) Output voltage (filled circles, solid line, blue axis) and available power density (hollow circles, dashed line, green axis) as a function of current from two further abiotically loaded devices (BG11 cell medium supplemented with 0.25 M NaCl).

Following 24 h of stabilization of the μBPV at open circuit potential, polarization and power curves (see **Figure** **3**) were generated by applying different resistance loads to the external circuit in the dark. In different devices, the abiotic peak power density outputs vary from around 0.2 to 1 W m^–2^ and were established at current densities of 1.5 and 3.5 A m^–2^, respectively. The large variation in device output between different devices stems from the variable position and shape of the cathode which is not lithographically defined in our current designs. Device improvements at this level may well provide a straightforward route to further improvement of the output power. Crucially, no major changes in current and power outputs were observed upon exposure to white light (see inset of Figure 2a).

### 2. 4. Comparison with Recent Literature

The exceptionally high power density in excess of 100 mW m^−2^ after subtraction of the abiotic background has been facilitated by the physical proximity of the cells to the anode allowing for operation without a proton-exchange membrane, which in turn leads to a low internal resistance in the device, as well as by the microscopic size of the anodic chamber allowing for a large ratio of active surface to volume. In macroscopic bio-electrochemical systems by contrast, parameters such as mass transport, reaction kinetics and ohmic resistance are expected to have detrimental effect on the electrical output.^4^,^29^

For a specific comparison, **Table** 1 gives an overview of the power densities as well as technical specifications of intrinsic BPVs (i.e., requiring no external energy) characterized in the recent literature, including an instance with an additional enzymatic cathode.^50^ While there are many aspects influencing the performance of a BPV, such as surface-to-volume ratio, photosynthetic organism, and electrode material, one can observe a trend that generally the mediator-free approaches surpass their counterparts that rely on electron mediators diffusing over large distances. It should be mentioned that many of the studies listed in Table1 were not intended to improve on output power. We also note that higher power densities have been observed^8^ when extrinsic energy was supplied.

**Table 1:** List of biophotovoltaic devices from the recent literature including previous microfluidic approaches that do not require additional energy input. The abbrevations used are anodic active area (AAA), anodic chamber volume (ACV), Nafion film over the cathodic chamber and Au cathode (N-Au), chemical sacrificial cathode (csc), carbon-platinum cathode impregnated on one side with Nafion (N-CPt), carbon paper coated with a thin layer of platinum (Pt-C), indium tin oxide (ITO), fluorine-doped tin oxide (FTO), carbon paint with polypyrrole (PPCP), carbon nanotubes on carbon paper (CNTCP), and benzoquinone (BQ). *Synechocystis TM* refers to mutant strains of the cyanobacterium *Synechocystis sp.* PCC 6803 where the three respiratory terminal oxidase complexes had been inactivated

Study	*P*_out_mW m^-2^	AAAmm^2^	ACVμL	Anode/cathode	Mediator	Photosynthetic organism
Chiao, 2006^23^	0.0004	50	4.3	Au/N-Au - csc	Methylene blue	*Anabaena sp.*
Bombelli, 2011^11^	1.2	80	150	ITO/N-CPt	K_3_[Fe(CN)_6_]	*Synechocystis sp.* PCC 6803
McCormick, 2011^14^	10	1300	12 600	ITO/Pt-coated glass	free	*Synechococcus sp*. WH 5701
Thorne, 2011^44^	24	230	2300	FTO/carbon cloth	K_3_[Fe(CN)_6_]	*Chlorella vulgaris*
Bombelli, 2012^45^	0.02	2000	20 000	ITO/Pt-C	free	*Oscillatoria limnetica*
Madiraju, 2012^46^	0.3	1500	60 000	Carbon fiber	free	*Synechocystis sp*. PCC 6803
Bradley, 2013^47^	0.2	1300	31–500	ITO/N-CPt	K_3_[Fe(CN)_6_]	*Synechocystis TM*
Lan, 2013^43^	13	4600	5 × 10^5^	Pre-treated graphite/csc	K_3_[Fe(CN)_6_]	*Chlamydomonas reinhardtii*
Lin, 2013^48^	10	2100	10^6^	Au mesh/graphite cloth	free	*Spirulina platensis*
Luimstra, 2013^49^	6	1400	70 000	PPCP/carbon cloth with Pt	free	*Pauschulzia pseudovolvox*
Sekar, 2014^50^	35	2.5	n/a	CNTCP/laccase on CNTCP	free	Nostoc sp.
Sekar, 2014^50^	100	2.5	n/a	CNTCP/laccase on CNTCP	BQ	Nostoc sp.
This study	105	0.03	0.4	InSnBi alloy/Pt	free	*Synechocystis sp*. PCC 6803

## 3. Discussion

In summary, we have described a microfluidic design for a mediator-less, membrane-free, bio-photovoltaic device. Electrical characterization of devices loaded with *Synechocystis sp*. PCC 6803 revealed peak power densities in excess of 100 mW m^–2^. In spite of the low power available per footprint area (currently of the order of 50 μW m^–2^) the promising performance and the simple fabrication process demonstrate the potential of our approach for generating biological solar cells with microfluidics.

Our approach is applicable to any photosynthetic organism forming biofilms. Furthermore, using the strategy presented in this work, further improvement of the power output should be readily achievable through reduction of the distance between anode and cathode and increase of the channel height. This flexibility in device geometry and the possibility of in situ electroplating of the anode underline the versatility of soft-lithography as a means for generating biophotovoltaic cells.

Options for enhanced miniaturization open pathways for the study of small cell cultures containing as little as tens of cells for rapid screening of electrochemically active microbes in the context of directed evolution.

## 4. Experimental Section

*Device Fabrication*: Devices were fabricated to a height of 25 μm using standard soft lithography^34^ for polydimethylsiloxane (PDMS) on glass. The designs include an array of 25 μm wide PDMS pillars spaced by 25 μm in order to allow for insertion of molten solder^36^,^37^ (Indalloy 19, Indium Corporation, Clinton NY, USA) on a hotplate set to 79 °C. Solidification of this InBiSn alloy upon removal from the heat yielded self-aligned wall electrodes using a single lithography step.^38^ This process is illustrated in Figure 1a,b. The cathode was constructed by inserting a strip of platinum wire of 100μm diameter through polyethylene tubing (Smiths Medical; 800/100/120; the same as used for contacting microfluidic devices in general) and sealing off both ends of the tubing with epoxy glue. Inserting this tube through a previously punched hole in the device generated a sealed electrical connection and is indicated by the orange wire (Pt) inside a white cylinder (tubing) in the scheme in Figure 1b. Note that this method for electrode fabrication also allowed for straightforward exchange of the cathode material, which would be beneficial for in situ electroplating the InBiSn alloy.

During settling and operation, the BPVs were oriented such that the bottom of the device was formed by the anode, and the glass slide as well as the pdms formed the side and top walls.

The total volume above the anode was below 400 nL, significantly reducing the consumption of biological material and chemicals of each experiment compared to macroscopic approaches.

*Electrode Area*: The accessible surfaces of these electrodes were ca. *A* ≈ 2.5 mm/2 × 25 μm ≈ 0.03 mm^2^ for the anode (only approximately one half of the total metal area was accessible due to the PDMS pillars) and of the order of 0.6 mm^2^ for the cathode, assuming the available length of the Pt wire to be 2 mm. Note that the majority of the cathode was inside the cavity of the insertion template. If one were to consider the entire horizontal cross-section of the device, the according area would double to 0.06 mm^2^, and the footprint of the device was at present around 60 mm^2^ including the access ports for fluid injection. This latter number can be reduced straightforwardly by more than one order of magnitude by redesigning the inlet ports.

*Cell Culture and Growth*: A wild-type strain of *Synechocystis sp*. PCC 6803 was cultivated from a laboratory stock.^11^ Cultures were grown and then analyzed in BG11 medium^51^ supplemented with 0.25 M NaCl. All cultures were supplemented with 5 mM NaHCO_3_ and maintained at 22 ± 2 °C under continuous low light (ca. 50 μmol m^−2^ s^−1^) in sterile conditions. Strains were periodically streaked onto plates containing agar (0.5–1.0%) and BG11 including NaCl, which were then used to inoculate fresh liquid cultures. Culture growth and density were monitored by spectrophotometric determination of chlorophyll content. Chlorophyll was extracted in 99.8% (v/v) methanol (Sigma-Aldrich, Gillingham, UK) as described previously.^52^

*Cell Injection and Settling*: First, the devices were filled with culture medium (BG11 with 0.25 M NaCl) and any air bubbles were removed by means of syringes attached via elastic polyethylene tubing (Smiths Medical; 800/100/120). *Synechocystis* cells suspended in BG11 (supplemented with NaCl) were then injected at a concentration of 100 μM chlorophyll. Maintaining the devices for 24 h at an orientation in which the metal alloy anode forms the bottom allows the cells to sediment on the electrode by gravity. This process creates a closely-spaced interface allowing the electrons to be transmitted to the anode (see Figure 1c,g) and thus favoring mediator-free operation. Throughout all experiments, the syringes are kept attached in order to prevent drying out of the BPV.

The complete device design used for the photolithography mask is presented in Figure 1d, and a microscopy image of a device colored with Coomassie blue is shown in Figure 1e. Furthermore, a picture of an array of devices is provided in the Supporting Information.

*Microfluidic BPV Measurement and Illumination*: In principle, the optimal way of extracting the voltage output of our biophotovoltaic device would be to determine the half-cell potentials individually by integrating reference electrodes into the devices. Since this is challenging in microfluidic devices,^53^ the terminal voltage of the BPV was measured instead, which did not offer insight into the potentials of the complex half-cell reactions but provided an accurate measurement for the power delivered to an external load.

Polarization curves were acquired by recording the terminal voltage *V* under pseudo-steady-state conditions^5^ with variable external loads (*R*_ext_) and plotting the cell voltage as a function of current density (current per unit anodic area). Typically, a time span of around 20 min was sufficient for a stable output (see Supporting Information Figure 2). The resistance values ranged from 24.8 MΩ to 324 kΩ (24.8, 13, 9.1, 5.3, 2.9, 1.1, 0.547, and 0.324 MΩ), where the internal resistance of the digital voltmeter of 100 MΩ has been taken into account. Voltages were recorded using an UT-70 data logger (Uni-Trend Limited, Hong Kong, China). The current delivered to the load was calculated from Ohm's law

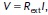
1and the power *P* is given by 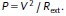
2

Based on the polarization curves, power curves were obtained for each system by plotting the power per unit area or power density *P*/*A* as a function of current density. These power density curves were further used to determine the average maximum power output for the microfluidic BPV system and the negative control. For all measurements, alligator clamps and copper wire served as connections to anode and cathode, and the temperature was kept at 22 ± 2 °C.

To characterize the light response, artificial light was provided by a warm white LED bulb (Golden Gadgets, LA2124-L-A3W-MR16), maintained at a constant output of 200 μmol m^−2^ s^−1^ at the location of the BPVs. A measured spectrum of the light source is shown in the Supporting Information. Light levels were measured in μmol m^−2^ s^−1^ with a SKP 200 Light Meter (Skye Instruments Ltd, Llandrindod Wells, UK).

The photoactive cells were illuminated through the glass slide forming the bottom of the device, resulting in an almost parallel angle of incidence on the cell layer. This geometry led to a decreased light intensity on the cells, which could be compensated for by using a more powerful light source in studies of photosynthetic materials or by altering the geometric arrangement of the devices when harnessing actual sunlight.

## Supporting Information

As a service to our authors and readers, this journal provides supporting information supplied by the authors. Such materials are peer reviewed and may be re-organized for online delivery, but are not copy-edited or typeset. Technical support issues arising from supporting information (other than missing files) should be addressed to the authors.
