# Supplementary material for: A High Power-Density, Mediator-Free, Microfluidic Biophotovoltaic Device for Cyanobacterial Cells
Source: Adv Energy Mater. 2014 Sep 16;5(2):1–6. doi: 10.1002/aenm.201401299 (PMC4503997; doi:10.1002/aenm.201401299)
Supplement: Supplementary file 1 — Supplementary [file aenm0005-0001-sd1.pdf]

Copyright WILEY-VCH Verlag GmbH & Co. KGaA, 69469 Weinheim, Germany, 2014.

ADVANCED ENERGY MATERIALS

Supporting Information

for *Adv. Energy Mater.,* DOI: 10.1002/aenm.201401299

A High Power-Density, Mediator-Free, Microfluidic Biophotovoltaic Device for Cyanobacterial Cells

Paolo Bombelli, Thomas Müller, Therese W. Herling, Christopher J. Howe, and Tuomas P. J. Knowles**

A High Power-Density Mediator-Free Microfluidic Biophotovoltaic Device for Cyanobacterial Cells

Paolo Bombelli,[1, a)] Thomas Müller,[2, a)] Therese W. Herling,[2] Christopher J. Howe,[1, b)] and Tuomas P. J. Knowles[2, c)]
[1)] *Department of Biochemistry, University of Cambridge, Tennis Court Road, Cambridge CB2 1QW, United Kingdom*
[2)] *Department of Chemistry, University of Cambridge, Lensfield Road, Cambridge CB2 1EW, United Kingdom*

I. DEVICE HANDLING

Figure 1 illustrates the operation of the device. After the fabrication of the device and the electrodes, the cell medium is injected using plastic syringes, and all air is removed by applying pressure on the fluid inlet and outlet. Thereafter, the elastic tubing is cut on one side and the cells are injected through another syringe. The syringes are then left attached to prevent drying of the device. Copper wires are soldered to the electrodes to provide electric connections. Finally, the device is positioned such that the anode forms the bottom and the cells sediment on it under the influence of gravity.

A magnified version of a device filled with a Coomassie blue solution is shown in Fig. 2(a), and a true-colour microscopy image of *Synechocystis sp.* PC 6803 cells settled on the alloy anode is presented in Fig. 1(b).

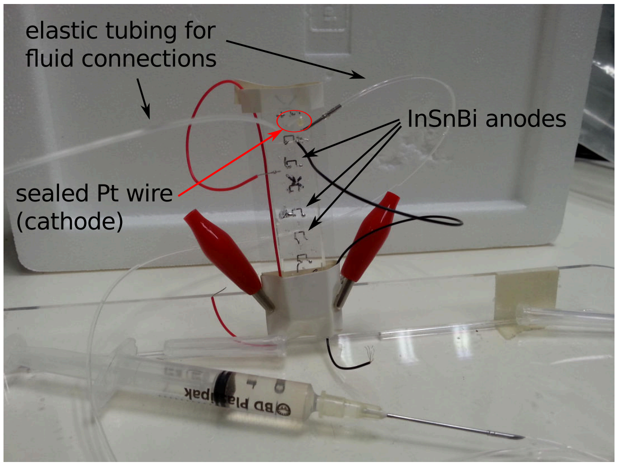

FIG. 1. Photograph of an array of devices. Anodes were fabricated for all cells, the sealed cathode and the fluid connectors are inserted into the topmost device.

[a)]These authors contributed equally to this work.
[b)]Electronic mail: ch26@cam.ac.uk
[c)]Electronic mail: tpjk2@cam.ac.uk

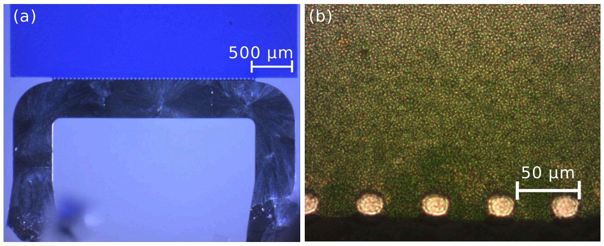

FIG. 2. (a) True-colour microscopy image of the indium alloy electrode and the fluid chamber filled with a Coomassie blue solution. (b) True-colour microscopy image of *Synechocystis sp.* PC 6803 cells settled on the electrode during 24 h.

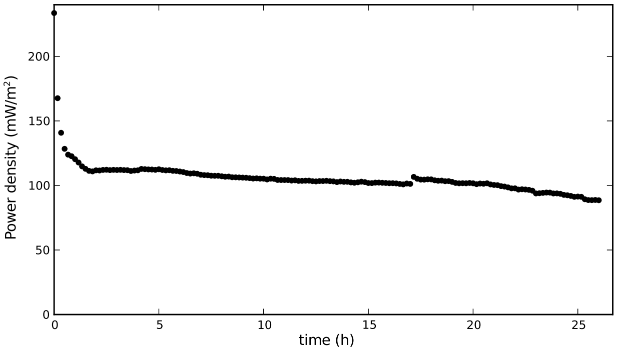

FIG. 3. Power output of an abiotically loaded device over an extended period of time, measured every 6 minutes.

II. DEVICE STABILITY

In order to assess the stability of our devices, a sample was loaded with cell medium and its power output was measured during more than 25 hours (see Fig. 3). After a sharp decrease in the first minutes, the device was stable for a period in excess of 24 hours.

III. ANODE MATERIAL

The low-melting point solder Indalloy 19 (Indium Corporation, Clinton NY, USA) - composed of 51% indium, 32.5% bismuth, and 16.5% tin - has been chosen due to its simplicity for generating self-aligned wall electrodes in microfluidic devices.[1–3] The melting point at 60 °C enables straightforward insertion into the device on a hot

plate at 79 °C, with the liquid metal patterned by polydimethylsiloxane pillars due to its surface tension. Upon removing the devices from the hot plate the alloy solidifies forming a solid electrode.

The standard potentials of each of the constituent metals are[4]

$$\mathrm{In} \leftrightarrow \mathrm{In}^{+} + e^{-} \qquad -0.13\ \mathrm{V}, \tag{1}$$

$$\mathrm{In} \leftrightarrow \mathrm{In}^{3+} + 3e^{-} \qquad -0.34\ \mathrm{V}, \tag{2}$$

$$\mathrm{Bi} \leftrightarrow \mathrm{Bi}^{3+} + 3e^{-} \qquad +0.32\ \mathrm{V}, \tag{3}$$

$$\mathrm{Sn} \leftrightarrow \mathrm{Sn}^{2+} + 2e^{-} \qquad -0.14\ \mathrm{V}. \tag{4}$$

These potentials are below the value for the oxidation of hydrogen to water at the cathode (+1.23 V), and therefore it is quite possible that oxides such as, for instance, In_2O_3, Bi_2O_3, $Bi_2Sn_2O_7$, or SnO_2 are forming on the anode. We have subtracted this oxidative current from our power estimates and did not see any significant deterioration in performance in a control over a time span of 25 hours (Fig. 3).

IV. ESTIMATES OF THE INTERNAL RESISTANCE

Measuring the voltage drop over an external resistor attached to a source yields the terminal voltage which is smaller than the actual cell voltage due to the internal resistance of the source

$$V_{\mathrm{terminal}} \equiv V = V_{\mathrm{cell}} - R_{\mathrm{int}} I = V_{\mathrm{cell}} - \frac{R_{\mathrm{int}}}{R_{\mathrm{ext}}} V. \tag{5}$$

Therefore,

$$V = \frac{V_{\mathrm{cell}}}{1 + R_{\mathrm{int}}/R_{\mathrm{ext}}}. \tag{6}$$

Since the IV-characteristics are not linear, the internal resistance of the cell, or - more likely - its output voltage, depends on the current drawn. Nevertheless, from the linear part of the polarisation curve at high currents (Fig. 2(a) in the main text) we can estimate the internal resistance to amount to

$$\frac{\Delta V}{\Delta I} \approx \frac{158 - 45\ \mathrm{mV}}{(4.4 - 1.7\ \mathrm{A/m^2}) * 0.03\ \mathrm{mm^2}} = 1.4\ \mathrm{M\Omega} \tag{7}$$

for the biotically loaded device and

$$\frac{\Delta V}{\Delta I} \approx \frac{130 - 30\ \mathrm{mV}}{(2.9 - 1.4\ \mathrm{A/m^2}) * 0.03\ \mathrm{mm^2}} = 2.2\ \mathrm{M\Omega} \tag{8}$$

for the abiotic control. Note that the internal resistance decreased by 1/3 with the addition of the cyanobacteria. Furthermore, since maximal power transfer to the external load is observed when the load resistance is matched to the internal resistance of the cell, we can double-check the above values by comparison to Fig. 2(b). There, the maximum power is observed for external resistances of 1.1 MΩ and 2.9 MΩ for biotic and abiotic filling, respectively. These values are in close agreement with the estimates from the polarisation curves.

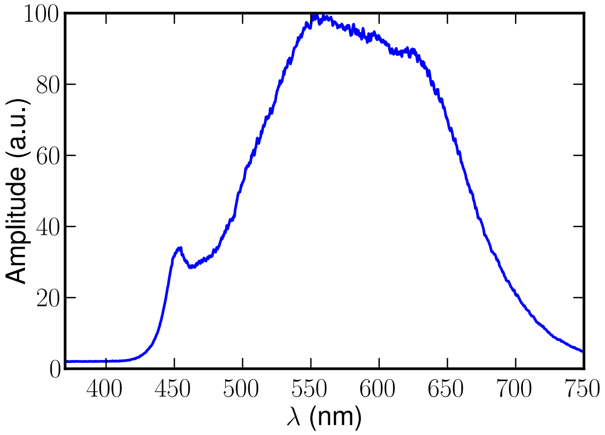

FIG. 4. Measured spectrum of the light source used in our experiments.

V. LIGHT SOURCE

In Fig. 4 we present the measured spectrum of the lamp we have used to illuminate our biophotovoltaic cells. From these data, we can also extract the weighted average wave number to be $10^7\ \mathrm{m}^{-1}$ which corresponds to a wavelength of around 570 nm. Therefore, the average energy per photon is 3.5×10^{-19} J, and the measured photon flux of 200 μmol/m^2/s yields an illumination intensity at the location of the devices of 42 W/m^2.

REFERENCES

[1] J.-H. So and M. D. Dickey, *Lab Chip*, 2011, **11**, 905–911.
[2] J. J. Li and C. M. Yip, *Biochimica et Biophysica Acta (BBA) - Biomembranes*, 2013, **1828**, 2272–2282.
[3] T. W. Herling, T. Müller, L. Rajah, J. N. Skepper, M. Vendruscolo and T. P. J. Knowles, *Appl Phys Lett*, 2013, **102**, 184102.
[4] *Standard Potentials in Aqueous Solution*, ed. A. J. Bard, R. Parsons and J. Jordan, CRC Press, 1985.
